# 765. Development of an Adaptable Roadmap for Implementation of the Infectious Diseases Society of America Core Antimicrobial Stewardship Curriculum in Infectious Diseases Fellowship Programs

**DOI:** 10.1093/ofid/ofad500.826

**Published:** 2023-11-27

**Authors:** Leila S Hojat, Payal K Patel, Priya Nori, Keith W Hamilton, Julie Ann Justo, Jennifer O Spicer, Ashleigh Logan, Kenza Bennani, Matthew S Lee, Chloe Bryson-Cahn, Erica J Stohs, Zachary Willis, Ryan P Moenster, Trevor C Van Schooneveld, Thea Brennan-Krohn, Cole Beeler, Amy Y Kang, Hawra Al Lawani, Kartik Cherabuddi, Gary Fong, Dilek Ince, Marisa Holubar, Molly L Paras, David Gaston, Rachel A Shnekendorf, Rostam Williams, Vera Luther

**Affiliations:** Case Western Reserve University/ University Hospitals Cleveland Medical Center, Cleveland, Ohio; Intermountain Health, Salt Lake City, Utah; Montefiore Health System, Bronx, NY; University of Pennsylvania Perelman School of Medicine, Philadelphia, PA; Dartmouth Hitchcock Medical Center; Emory University School of Medicine, Atlanta, GA; IDSA, Arlington, Virginia; IDSA, Arlington, Virginia; Beth Israel Deaconess Medical Center, Boston, Massachusetts; Harborview Medical Center, Seattle, Washington; University of Nebraska Medical Center, Omaha, Nebraska; University of North Carolina School of Medicine, Chapel Hill, NC; St. Louis College of Pharmacy at UHSP/VA St. Louis HCS, St. Louis, Missouri; University of Nebraska Medical Center, Omaha, Nebraska; Beth Israel Deaconess Medical Center, Boston, Massachusetts; Indiana University School of Medicine, Indianapolis, IN; Chapman University School of Pharmacy, Irvine, CA, Irvine, California; Beth Israel Deaconess Medical Center, Boston, Massachusetts; University of Florida, Gainesville, Florida; Chapman University School of Pharmacy, Irvine, California; University of Iowa Hospitals & Clinics, Iowa City, Iowa; Stanford University School of Medicine, Stanford, CA; Massachusetts General Hospital, Harvard Medical School , Boston, MA; Vanderbilt University Medical Center, Nashville, Tennessee; Infectious Diseases Society of America, Arlington, Virginia; Infectious Diseases Society of America, Arlington, Virginia; Wake Forest University School of Medicine, Winston Salem, NC

## Abstract

**Background:**

The Infectious Diseases Society of America (IDSA) developed and disseminated a Core Antimicrobial Stewardship (AS) Curriculum intended to formalize AS training in infectious diseases (ID) fellowship programs in 2018. This study identified individual program approaches to curriculum implementation and intended to use this information to develop an implementation guide tailored to specific program needs.

**Methods:**

We distributed surveys to all fellowship program directors (PDs) who had previously implemented the Core AS Curriculum. Questions were designed to identify ID program structure, curriculum participants, curriculum sections and materials utilized, and resources and barriers to implementation. Both structured and qualitative responses were captured. The results were summarized descriptively and organized into a framework connecting barriers to proposed solutions.

**Results:**

PDs from 34 unique programs who had administered the Core Curriculum to an estimated 405 ID fellows responded to the survey, out of the 159 institutions invited (21.4%). Most represented adult programs which had administered the curriculum for at least 2 years (**Table 1**). Additional learners often included ID faculty and pharmacy trainees, and teachers were mostly AS program leadership. Most PDs reported limited faculty time as a barrier to implementation, whereas dedicated AS curricular time was a resource available to most programs (**Figure 1**). Approaches to curriculum implementation based on survey responses relating to each fellowship program feature were suggested, some of which applied to multiple program features (**Figure 2**). Qualitative feedback was generally positive, and most PDs indicated that they intended to continue to implement the curriculum. Additional materials such as a facilitator guide and demonstrations were proposed as other components which could assist with curriculum implementation.

**Table 1.**
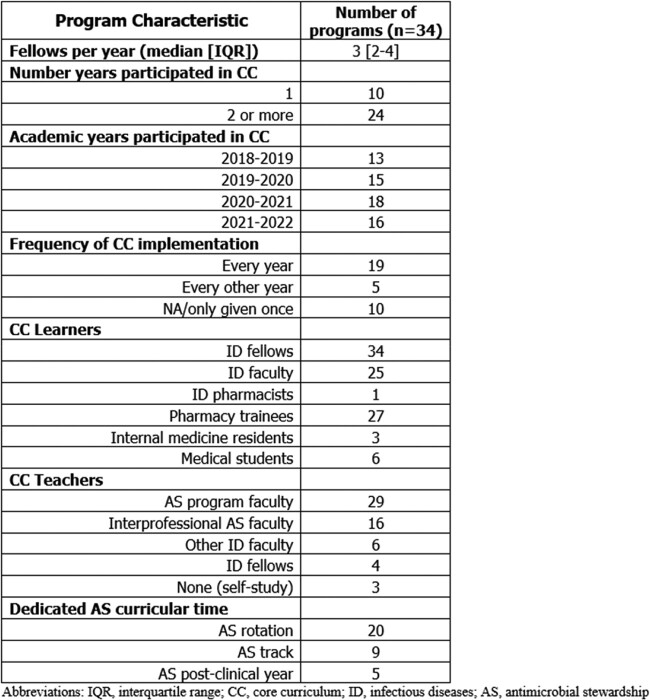
Descriptive characteristics of infectious diseases fellowship programs participating in the survey.

**Figure 1.**
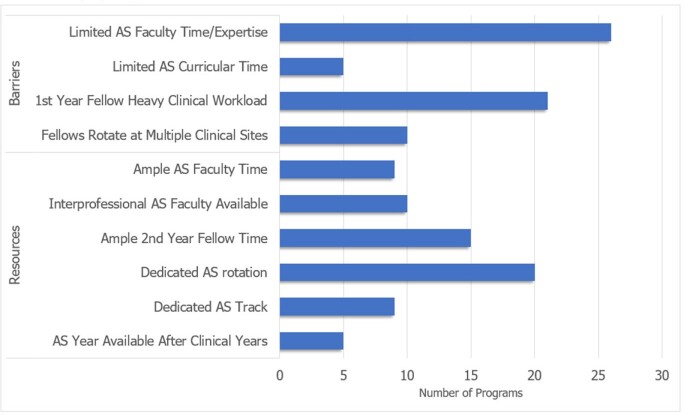
Self-reported barriers and resources described within each infectious diseases fellowship program.

**Figure 2.**
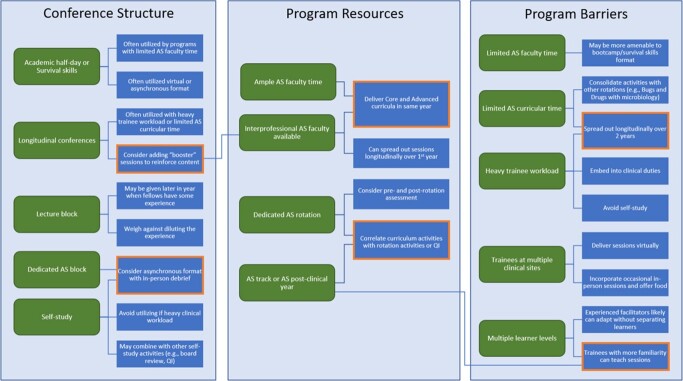
Roadmap for infectious diseases fellowship program features.

Roadmap with infectious diseases fellowship program features (rounded green boxes) connected to identified potential approaches to curriculum implementation (rectangular blue boxes). Program features are categorized as relating to conference structure, program resources, or program barriers. Suggested approaches with potential application to multiple program features are highlighted in orange.

**Conclusion:**

The IDSA Core AS curriculum provides an effective means of formalizing basic AS education into ID fellowship training. Curriculum implementation can be optimized by tailoring to training program resources and unique features. An implementation roadmap may be a useful tool to assist ID fellowship PDs with this task.

**Disclosures:**

**Payal K. Patel, MD MPH**, qiagen: Honoraria **Julie Ann Justo, PharmD, MS, FIDSA, BCPS**, Gilead Sciences: Advisor/Consultant|Shionogi: Advisor/Consultant|Vaxart: Stocks/Bonds **Erica J. Stohs, MD, MPH**, bioMerieux: Grant/Research Support|Merck: Grant/Research Support **Zachary Willis, MD, MPH**, Merck Sharp & Dohme Corp: Grant/Research Support|Pfizer Inc: Grant/Research Support **Trevor C. Van Schooneveld, MD, FSHEA, FACP**, AN2 Therapeutics: Grant/Research Support|Biomeriuex: Advisor/Consultant|Biomeriuex: Grant/Research Support|Insmed: Grant/Research Support|Thermo-Fischer: Honoraria **Amy Y. Kang, Pharm.D., BCIDP**, Paratek: Grant/Research Support **Kartik Cherabuddi, MD, FACP, FIDSA**, Contrafect Corporation: Grant/Research Support|Labcorp Drug Development: Grant/Research Support|Merck Sharp & Dohme: Grant/Research Support **Gary Fong, PharmD**, Critical Innovations, LLC: Advisor/Consultant **Molly L. Paras, MD**, Angiodynamics: Honoraria **David Gaston, MD PhD**, American Association of Clinical Chemistry: Honoraria|BioMerieux, Inc: Advisor/Consultant|IDbyDNA, Inc.: Grant/Research Support|Illumina, Inc.: Grant/Research Support

